# Integrated Analysis of Gut Microbiota and Metabolome Profiling Reveals the Effects of Feeding Approaches on Infants' Gut Microenvironment

**DOI:** 10.1002/fsn3.71777

**Published:** 2026-04-14

**Authors:** Simin Zhu, Wenjuan Tu, Wenting Zhang, Li Qing, Chengan Wang

**Affiliations:** ^1^ Center for Children's Health Promotion Affiliated Changzhou Children's Hospital of Nantong University Changzhou Jiangsu China; ^2^ Central Laboratory of Pediatrics Affiliated Changzhou Children's Hospital of Nantong University Changzhou Jiangsu China; ^3^ School of Stomatology Nanjing Medical University Nanjing Jiangsu China

**Keywords:** arachidonic acid metabolism, breastfeeding, gut microbiota, immune tolerance, metabolites

## Abstract

Breast milk can improve infant health by exerting beneficial effects on the developing gut microbiota and metabolites. However, infant formula composition undergoes continual refinement to better approximate breast milk. This study aimed to timely monitor the evolving disparities in the gut microenvironment between breastfed (BF) and formula‐fed (FF) infants. This prospective cohort study enrolled 44 healthy infants at 6 months of age, stratified by infant feeding modality into BF (*n* = 27) and FF (*n* = 17) groups. Fecal samples of all infants were collected and tested. Fecal microbiota was tested via full‐length 16S rRNA sequencing, and fecal metabolites were tested via untargeted UPLC‐MS/MS metabolomics. Associations between gut microbiota and metabolites were evaluated using Spearman's rank correlation test. Bacterial full‐length 16S rRNA gene sequencing analysis revealed that Escherichia, Staphylococcus, Haemophilus, and Rothia were more enriched in the BF group, but Enterococcus and Mediterraneibacter were more enriched in the FF group conversely (*p* < 0.05). UPLC‐MS/MS metabolomic profiling revealed 600 dysregulated metabolites (*p* < 0.05) discriminating BF and FF infants, with 225 metabolites up‐regulated and 375 metabolites down‐regulated in the BF group. Further analysis of the significantly differential metabolites of the top 100 Variable Importance in Projection (VIP) revealed that 12 metabolites could be mapped onto Kyoto Encyclopedia of Genes and Genomes database (KEGG) pathways, in which the metabolites were mostly mapped onto Arachidonic acid (AA) metabolism and were down‐regulated in the BF group, including Thromboxane B2 (TXB2), 5‐hydroxyeicosatetraenoid acid (HETE), (15S)‐Prostaglandin A2 (PGA2), and 11‐dehydro‐TXB2. Breastfeeding establishes a distinct ecological imprinting on the infant gut, promoting specific taxa and downregulating pro‐inflammatory fecal AA metabolites. These disparities suggest breastfeeding uniquely modulates the microbial‐metabolic axis to maintain a lower inflammatory tone, offering immunological advantages despite modern formula innovations.

## Graphical abstract

This graphical abstract illustrates the gut microenvironment disparities between breastfed and formula‐fed infants at 6 months of age. Through multi‐omics analysis, breastfeeding was shown to establish a distinct ecological imprinting characterized by favorable microbial shifts and down‐regulated pro‐inflammatory arachidonic acid metabolism, thereby maintaining a lower inflammatory tone and providing unique immunological advantages.

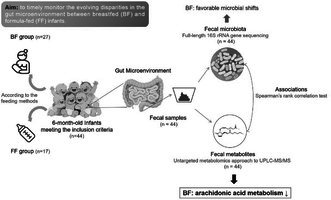

## 1 Introduction

Exclusive breastfeeding is endorsed by WHO as the optimal nutritional standard during the first 6 months of life. Both human milk and infant formula serve as sole‐source nutrition in early infancy, exerting profound programming effects on lifelong health outcomes (Beaudry et al. 1995; Fujimura et al. 2016; Hanson et al. 2003; Melnik et al. 2016). Breastfeeding confers significant protection against diarrheal and respiratory infections, while attenuating the risk of inflammatory disorders (Victora et al. 2016). While modern infant formulas are fortified to approximate the macronutrient and micronutrient profiles required for infant growth—including bioactive additives such as human milk oligosaccharides (HMOs), probiotics, lactoferrin, galacto‐/fructo‐oligosaccharides (GOS/FOS), and optimized lipid matrices—they remain functionally distinct from human milk due to the absence of numerous immunomodulatory, epigenetic‐regulating, and microbiota‐shaping components unique to the lactational niche (Blesa‐Baviera et al. 2025; Brink et al. 2020; Oozeer et al. 2013). The bioactive constituents of breast milk have the potential to influence the initial colonization and subsequent development of the infant gut microbiota, thereby potentially impacting health outcomes (Brink et al. 2020).

The gut microbiota serves as a fundamental biological determinant orchestrating infant development and lifelong health, functionally regulating host metabolic processes and immune maturation through microbiome‐host crosstalk (Chen et al. 2023). Infancy constitutes a critical window for gut microbiome assembly, with enduring consequences for lifelong health trajectories (Davis et al. 2022). Following a simplified but generalizable successional paradigm, facultative anaerobes (Staphylococcus, Streptococcus, Enterobacteriaceae, Lactobacillus) function as pioneering colonists that establish the reducing microenvironment essential for subsequent obligate anaerobe dominance—notably *Bifidobacterium, Clostridium*, and *Bacteroides*—through oxygen consumption (Davis et al. 2017; Rodríguez et al. 2015).

The gut microbiota does not exist and function in isolation, but rather interacts with gut metabolites, which is highly significant in infant health. In early life, short chain fatty acids (SCFAs) biosynthesis could be driven by *Bifidobacterium* and *Bacteroides* through specialized fermentation of human milk oligosaccharides (HMOs), which participate in immune programming (Nguyen et al. 2021).

Firmicutes and Bacteroidetes mediate the microbial transformation of bile acids, regulating host lipid metabolism (Xiong et al. 2022). *Bifidobacterium* metabolizes indole‐3‐lactic acid (ILA) to elicit cytoprotection in gut epithelia via coordinated AhR and Nrf2 pathway activation (Ehrlich et al. 2020). Metabolomics provides a robust framework for elucidating the interplay between the gut microbiota and host metabolism (Chen et al. 2023). These metabolites may come directly from the nutritional and non‐nutritional environment by microbes (Bruce et al. 2023; Sillner et al. 2021). Consequently, gut metabolites constitute equally critical functional mediators as the gut microbiota itself in deciphering microbe‐host crosstalk and physiological impact (Brink et al. 2020). Additionally, given the continuous refinement of infant formula composition, proactive monitoring of evolving disparities in gut microenvironment between BF and FF infants remains imperative. Hence, we established a clinical cohort of healthy Chinese infants aged 6 months, which were divided into BF group and FF group according to the feeding methods. Fecal samples of infants from the two groups were collected and tested. Full‐length 16S rRNA gene sequencing was used to detect fecal microbiota, and an untargeted metabolomics approach to UPLC‐MS/MS was used to detect fecal metabolites.

## 2 Materials and Methods

### 2.1 Subjects Selection

In this study, 6‐month‐old infants from the pediatric health department of Affiliated Changzhou Children's Hospital of Nantong University from January 2024 to August 2024 were selected according to the inclusion criteria and divided into BF group and FF group based on their feeding patterns. All the infants had not yet added complementary food. To ensure accuracy, exclusive feeding status (only breast milk or formula) was verified at enrollment through structured interviews and 3‐day dietary records provided by the caregivers. They were healthy full‐term infants, free from congenital defects, genetic disorders, acute diseases, and had normal weight. To mitigate potential confounding from early‐life medical interventions, we excluded: (a) infants receiving antibiotics or probiotics postnatally; (b) mothers using antimicrobial agents, probiotic formulations, or prebiotic supplements within 30 days antepartum; and (c) infants born to parents with chronic cardiometabolic, autoimmune, or endocrine disorders.

### 2.2 Sample Collection

Fecal samples were collected from infant diapers, immediately flash‐frozen at −20°C, and transferred to −80°C cryogenic storage. All 44 samples underwent single‐thaw processing for downstream analyses, maintaining strict cold‐chain integrity to preserve microbial and metabolic integrity.

### 2.3 16S Ribosomal RNA Gene Sequencing

Genomic DNA was extracted from fecal samples using the FastPure Stool DNA Isolation Kit (MJYH Biotech, Shanghai, China) following manufacturer protocols. DNA integrity was verified by 1% agarose gel electrophoresis, while concentration and purity were quantified via NanoDrop 2000 UV–vis spectrophotometry (Thermo Scientific, Wilmington, USA). The V1‐V9 hypervariable regions of bacterial 16S rRNA genes were amplified with universal bacterial primers 27F (5′‐AGRGTTYGATYMTGGCTCAG‐3′) and 1492R (5′‐RGYTACCTTGTTACGACTT‐3′). PCR amplicons underwent purification with AMPure PB beads (Pacific Biosciences) followed by quantification on a Synergy HTX platform (BioTek). Full‐length sequencing was conducted via PacBio Sequel IIe system (Pacifc Biosciences, CA, USA) in Majorbio Bio‐Pharm Technology Co. Ltd. (Shanghai, China). Subsequent bioinformatic processing using SMRTLink v11.0 yielded high‐fidelity (HiFi) reads.

Operational taxonomic units (OTUs) were delineated from HiFi reads at a 97% similarity threshold via USEARCH 11, with dominant sequences per OTU designated as representatives. Taxonomic assignment of representative sequences was executed using the RDP Classifier (v2.13) against the 16S rRNA database with a 0.7 confidence threshold.

Bioinformatic analysis of the gut microbiota was carried out using the Majorbio Cloud platform (https://cloud.majorbio.com). Based on the OTUs information, alpha diversity indices including the observed richness (Sobs), ACE, Chao1, Shannon and Simpson were calculated with Mothur v1.30.2, and the differences between these indices between different groups were analyzed by Wilcoxon rank‐sum test. The similarity between the microbial communities in different samples was determined by principal coordinate analysis (PCoA) based on Bray–curtis dissimilarity using Vegan package (version 2.4.3). The PERMANOVA test was used to assess the percentage of variation explained by the treatment along with its statistical significance using Vegan package (version 2.4.3). Additionally, Relative abundance of the respective predominant phylum in different groups was analyzed with R (Version 3.3.1). Differential abundance testing between different groups employed Wilcoxon rank‐sum tests to identify significant differences in microbial composition at both the phylum and genus levels.

### 2.4 Untargeted Metabolomics Analyses

A 50 mg (wet weight, ±0.5 mg) fecal sample was combined with 400 μL of extraction solution (methanol: water = 4:1, v: v) containing 0.02 mg/mL of the internal standard L‐2‐chlorophenylalanine. The mixture was processed in a high‐throughput tissue homogenizer (Wonbio‐96c, Shanghai Wanbo Biotechnology Co. Ltd.) for 6 min at −10°C and 50 Hz.

The homogenate underwent cryoprecipitation at −20°C (30 min), followed by centrifugation (13,000 × *g*, 4°C, 15 min). Resultant supernatants were aliquoted into LC‐MS/MS autosampler vials for chromatographic separation. Quality Control (QC) samples were prepared by pooling an equal volume of the extract from each individual test sample to monitor the stability of the instrument and the reproducibility of the extraction process. QC replicates underwent identical processing and analytical procedures as study samples. These were strategically bracketed throughout analytical batches (every 5–15 injections) to validate system stability.

LC‐MS/MS profiling was performed using a SCIEX UPLC‐Triple TOF 5600 platform (Shanghai Majorbio Bio‐Pharm Technology Co. Ltd.) fitted with an ACQUITY HSS T3 column (100 × 2.1 mm, 1.8 μm; Waters, USA). The chromatographic separation implemented a binary solvent system: (A) acidified water (0.1% formic acid): acetonitrile (95:5, v/v) and (B) acidified acetonitrile (0.1% formic acid): isopropanol: water (47.5:47.5:5, v/v). Operational parameters included: 0.40 mL/min flow rate, 40°C column temperature, and 10 μL injection volume.

The mass spectrometric data were analyzed by positive and negative electrospray ionization. Instrumental parameters were optimized as follows: Ion source at 550°C; curtain gas (CUR, 30 psi); nebulizer gases (GS1/GS2, 50 psi each); ion‐spray voltage floating (ISVF) at −4000 V in negative mode and 5000 V in positive mode, respectively; declustering potential 80 V; collision energy (CE) ramped 20–60 eV for MS/MS scans. Data acquisition employed Information Dependent Acquisition (IDA) mode covering m/z 50–1000 for comprehensive metabolite characterization.

The pretreatment of LC/MS raw data was performed by Progenesis QI v3.0 (Waters Corporation, Milford, USA) software. At the same time, the metabolites were identified by searching database, and the main databases were the Human metabolome database (HMDB) and Majorbio Database. The data were preprocessed using the Majorbio Cloud Platform, a freely accessible online resource (https://cloud.majorbio.com). Following data preprocessing, multivariate statistical analyses, including Principal Component Analysis (PCA) and Orthogonal Partial Least Squares Discriminant Analysis (OPLS‐DA), were conducted utilizing the R package “ropls” (version 1.6.2). To assess model robustness and prevent overfitting, the OPLS‐DA model was validated using 200‐time permutation tests. The *p*‐value for the permutation test was defined as the ratio of the number of random models with higher accuracy than the original model to the total number of permutations. A *p* < 0.05 was considered to indicate an optimal and statistically significant model.

Significantly different metabolites were identified as those with VIP > 1, *p* < 0.05. These criteria were derived from the VIP values obtained through the OPLS‐DA model and the *p* values calculated using the Wilcoxon rank‐sum test. To control the false‐positive rate inherent in high‐dimensional omics data, we have used the Benjamini‐Hochberg (BH) method to calculate the False Discovery Rate (FDR) adjusted *p*‐values (*p_adjust*). Metabolite identification was performed following the MSI guidelines for Level 2 identification. Two sub‐levels of confidence were assigned: Level B(i), representing an exact match against our in‐house experimental MS/MS library; and Level B(ii), representing an exact match against public or theoretical MS/MS libraries. The differential metabolites between the two groups were subsequently mapped to their respective biochemical pathways via metabolic enrichment and pathway analysis, utilizing the KEGG database as the reference library.

### 2.5 Spearman Correlation Coefficient Model

Spearman correlations between genus‐level microbiota and metabolites were computed using R (version 1.6.2), with heatmap visualization generated via the pheatmap package (version 3.3.1).

### 2.6 Statistics Analysis of Participant Characteristics

Statistical analyses were conducted using IBM SPSS Statistics 26.0. Continuous variables with normal distribution are presented as mean ± standard deviation (SD), with between‐group comparisons assessed by independent Student's *t*‐tests. Non‐normally distributed continuous variables are reported as median (interquartile range), analyzed via Wilcoxon rank‐sum tests. Categorical variables are presented as counts (percentages), with intergroup differences assessed using Chi‐square or Fisher's exact tests, as appropriate. A two‐tailed *p* value of less than 0.05 was considered statistically significant.

## 3 Results

### 3.1 Participant Characteristics

We profiled gut microbiota and metabolites in 44 healthy infants, comparing breastfeeding (BF, *n* = 27) versus formula feeding (FF, *n* = 17). In the FF group (*n* = 17), 15 infants were fed cow's milk‐based formulas, while 2 received amino acid‐based formulas (AAF). The two AAFs were free of prebiotic additives. Among the 15 cow's milk‐based formulas, prebiotic fortification included HMOs (*n* = 1, containing 2′‐fucosyllactose, lacto‐N‐neotetraose, 3‐fucosyllactose, 6′‐sialyllactose, and 3′‐sialyllactose), GOS + FOS (*n* = 5), GOS only (*n* = 8), and FOS only (*n* = 1). All FF infants consumed formulas fortified with AA in the range of 0.33%–0.61% (as a percentage of total fat). All mother‐infant dyads met stringent exclusion criteria: no maternal antibiotic or probiotic exposure during pregnancy or delivery, and no postnatal antimicrobial or probiotic administration to infants. Table 1 presents the key characteristics of the participants enrolled in this study. At enrollment (6 months of age), the mean weight and height of the infants were 8.05 ± 1.17 kg and 67.11 ± 2.69 cm, respectively. The cohort (*n* = 44) was 47.7% male, and the rate of vaginal delivery was 47.7%. Statistical comparisons did not reveal any significant differences in the baseline characteristics (*p* > 0.05).

**TABLE 1:** Comparison of participant characteristics between BF and FF infants.

Characteristics	BF (*n* = 27)	FF (*n* = 17)	*t/Z/X* ^*2*^	*p*
Maternal age (year)	29.00(27.00, 31.00)	29.00(27.50, 33.50)	−0.364^a^	0.716
Gestational age (weeks)	39.16 ± 1.23	39.42 ± 0.95	−0.750^b^	0.457
Infant weight (kg)	7.95 ± 1.28	8.21 ± 0.98	−0.708^b^	0.483
Infant height (cm)	66.96 ± 2.83	67.36 ± 2.52	−0.476^b^	0.637
Baby gender, *N* (%)				
Male	12(44.40)	9(52.90)	0.302^c^	0.583
Female	15(55.60)	8(47.10)		
Delivery methods, *N* (%)				
Vaginal	12(44.40)	9(52.90)	0.302^c^	0.583
Cesarean	15(55.60)	8(47.10)		
Parity, *N* (%)				
1	20(74.10)	14(82.40)		0.716
2	7(25.90)	3(17.60)		

*Note:* ^a^means *Z* value, ^b^means *t* value, ^c^means *X* ^*2*^ value, test value which is not marked means Fisher's exact test.

### 3.2 Comparison of Gut Microbiota Between Feeding Groups

Fecal microbiota composition was characterized via full‐length 16S rRNA gene sequencing. We investigated the fecal microbiota of all samples. Full‐length sequencing yielded 804,936 quality‐filtered 16S rRNA reads across 44 samples. Following rarefaction to an even depth of 18,294 reads per sample and 97% identity clustering, we identified 196 OTUs, ranging from 14 to 57 OTUs per sample.

**FIGURE 1:**
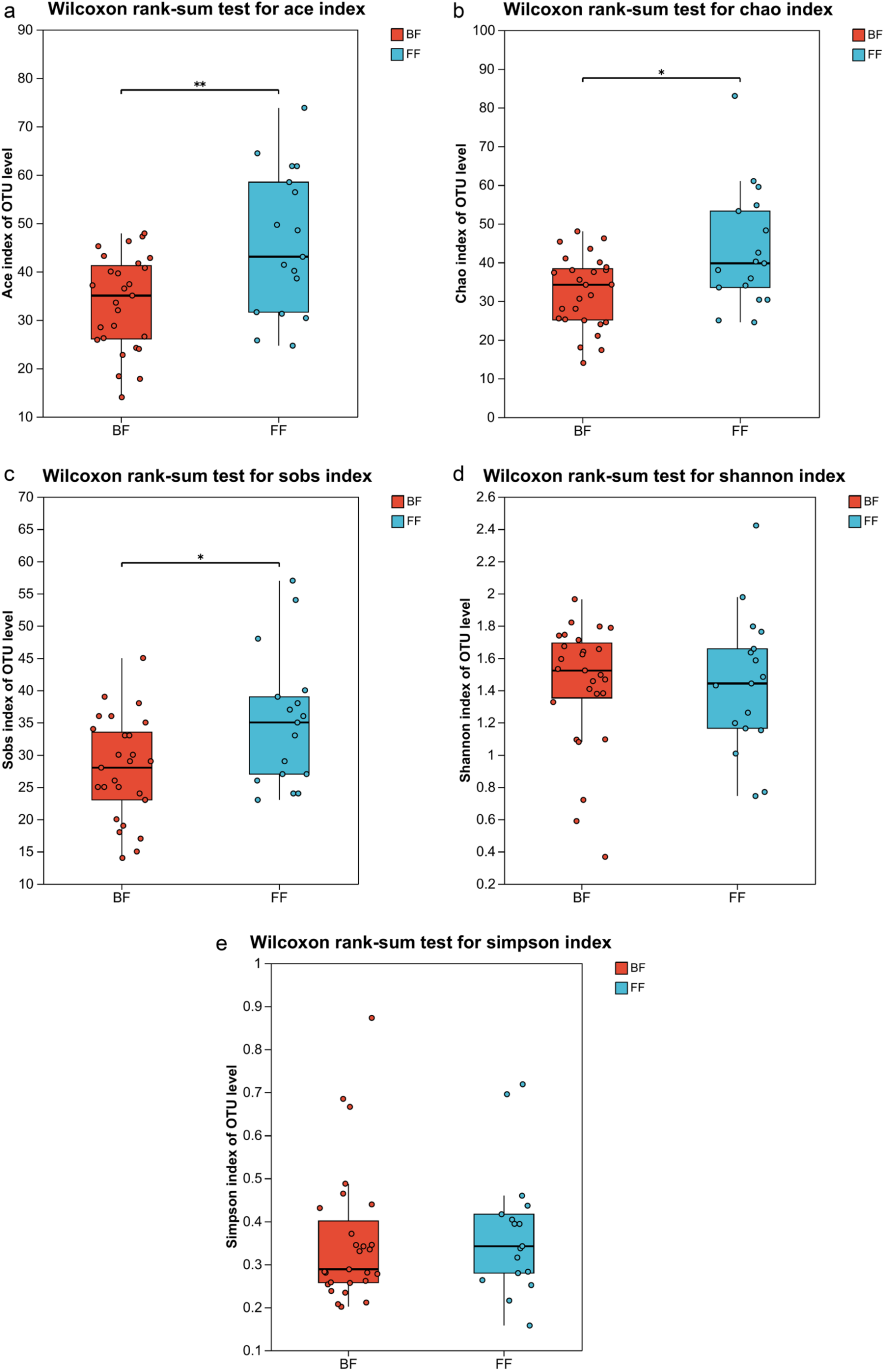
Comparison of alpha diversity indices between the two groups. (a–c) ACE, Chao and Sobs indices represent the community richness. (d, e) Shannon and Simpson indices represent the community diversity. The *p* value was calculated by Wilcoxon rank‐sum test. *p < 0.05; **p < 0.01.

Feeding methods were strongly associated with the alpha diversity indices of gut microbiota (Figure 1). ACE, Chao, Sobs, Shannon, and Simpson indices were calculated to assess the alpha diversity representing the community richness and diversity. Comparative analysis revealed significantly reduced microbial richness in BF infants versus FF counterparts, evidenced by: ACE index (*p* = 0.009), Chao index (*p* = 0.025), and Sobs index (*p* = 0.026). In contrast, the diversity showed no significant intergroup differences: Shannon index (*p* = 0.791) and Simpson index (*p* = 0.455).

**FIGURE 2:**
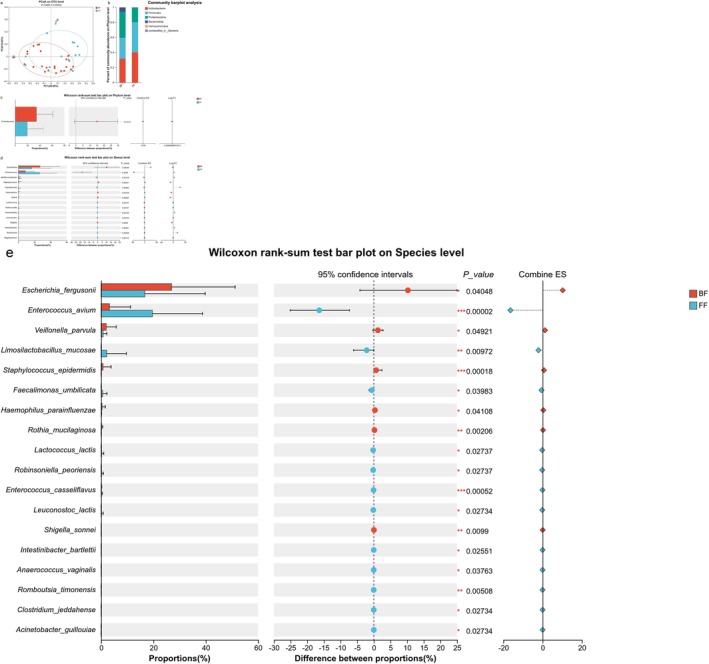
Microbial community structure of infant stool in the two groups. (a) The principal coordinate analysis (PCoA) result revealed a significant difference in gut bacterial structure between the two groups (beta diversity). The two principal component scores accounted for 28.56 (PC1) and 16.83% (PC2) of total variations, respectively. Each point represents an individual sample. Ellipses represent the 95% confidence interval. The *p* value was calculated by PERMANOVA. (b, c) Relative abundance and comparison of community composition at the phylum level (Wilcoxon rank‐sum test). (d) Comparison of community composition at the genus level (Wilcoxon rank‐sum test). (e) Comparison of community composition at the species level (Wilcoxon rank‐sum test). *p < 0.05; **p < 0.01; ***p < 0.001.

PCoA of beta diversity revealed a trend toward structural segregation between the two groups, approaching statistical significance (*p* = 0.054, *R* = 0.088) as shown in Figure 2a. Significant disproportionality in microbial abundances was observed across taxonomic levels between feeding cohorts. Actinobacteria, Firmicutes, Proteobacteria, and Bacteroidetes constituted the dominant bacterial phyla in each group (Figure 2b). At the phylum level, Proteobacteria (34.585%) dominated the BF group, whereas Actinobacteria (40.138%) predominated in the FF group. What's more, the community abundance of Proteobacteria was significantly higher in the BF group compared with the FF group (*p* = 0.012) (Figure 2c). At the genus level (Figure 2d), *Escherichia, Staphylococcus, Haemophilus, Rothia*, and *Shigella* were more enriched in the BF group. Conversely, *Enterococcus, Mediterraneibacter, Faecalimonas, Lactococcus, Robinsoniella, Granulicatella, Leuconostoc, Intestinibacter, Romboutsia*, and *Mogibacterium* were more enriched in the FF group (*p* < 0.05). At the species level (Figure 2e), *Escherichia_fergusonii, Veillonella_parvula, Staphylococcus_epidermidis (S. epidermidis), Rothia_mucilaginosa, Haemophilus_parainfluenzae*, and *Shigella_sonnei* were more enriched in the BF group. Conversely, *Enterococcus_avium, Limosilactobacillus_mucosae, Faecalimonas_umbilicata, Lactococcus_lactis, Robinsoniella_peoriensis, Enterococcus_casseliflavus, Leuconostoc_lactis, Intestinibacter_bartlettii, Anaerococcus_vaginalis, Romboutsia_timonensis, Clostridium_jeddahense*, and *Acinetobacter_guillouiae* were more enriched in the FF group (*p* < 0.05).

### 3.3 Comparison of Gut Metabolites Between Feeding Groups

**FIGURE 3:**
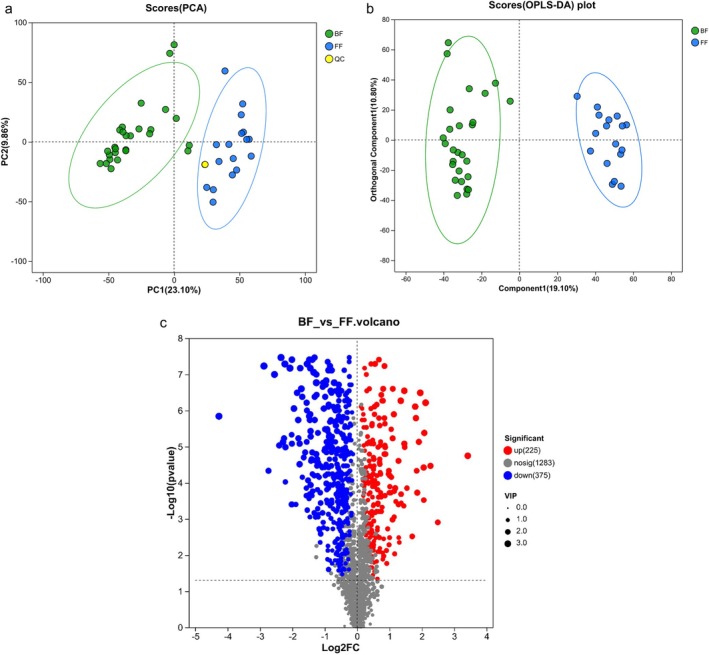
Differential metabolic profiles of infant stool between the two groups. (a, b) Principal Component Analysis (PCA) and Orthogonal Partial Least Squares Discriminant analysis (OPLS‐DA) score plots reveal separation in metabolite profiles between the two groups. Each point represents an individual sample, except the yellow one, which represents the quality control (QC). Ellipses represent the 95% confidence interval. (c) Volcano plot. Each point represents a specific metabolite. The size of the point represents the variable importance (VIP). Red points represent significantly up‐regulated metabolites, blue points represent significantly down‐regulated metabolites, and gray points are metabolites with no significant difference. The points closer to the left, right, and top edges show more significant differences in expression.

Fecal metabolomic profiling was conducted using UPLC‐MS/MS. Following rigorous data processing—including peak filtering, missing value imputation, and probabilistic quotient normalization—a total of 10,879 m/z features were retained (positive mode (POS): 5603; negative mode (NEG): 5276). Integrated POS‐NEG datasets were subjected to multivariate analysis. PCA demonstrated exceptional analytical stability, evidenced by tight clustering of QC samples in the score plot (Figure 3a), with the first two principal components explaining 32.96% cumulative variance (PC1 = 23.10%, PC2 = 9.86%). To more specifically model differences between the two groups, OPLS‐DA was used, with the first two components accounting for only ~30% of total variance. Both plots of PCA and OPLS‐DA revealed distinctive classification of the two groups (Figure 3a,b), with the BF group far from the FF group. OPLS‐DA demonstrated high interpretive and predictive power, with R^2^Y(cum) = 0.962 and Q ^2^(cum) = 0.896 (Table S1). Validation through 200‐time permutation tests confirmed that the model was not overfitted, as the *p*‐values for both R^2^Y and Q^2^ were significantly less than 0.05 (Figure S1). The results indicate that feeding methods altered the metabolites. To identify metabolites driving intergroup separation, VIP scores were computed based on the OPLS‐DA. Metabolite features with VIP > 1.0 (*p* < 0.05) were prioritized as key discriminators, where higher VIP values denote stronger contributions to class segregation. Multivariate analysis identified 600 differentially abundant metabolites (VIP > 1.0, *p* < 0.05, Table S2) distinguishing BF and FF groups, visualized in the volcano plot (Figure 3c). Among these, 225 metabolites demonstrated significant upregulation in BF infants, while 375 exhibited downregulation.

**FIGURE 4:**
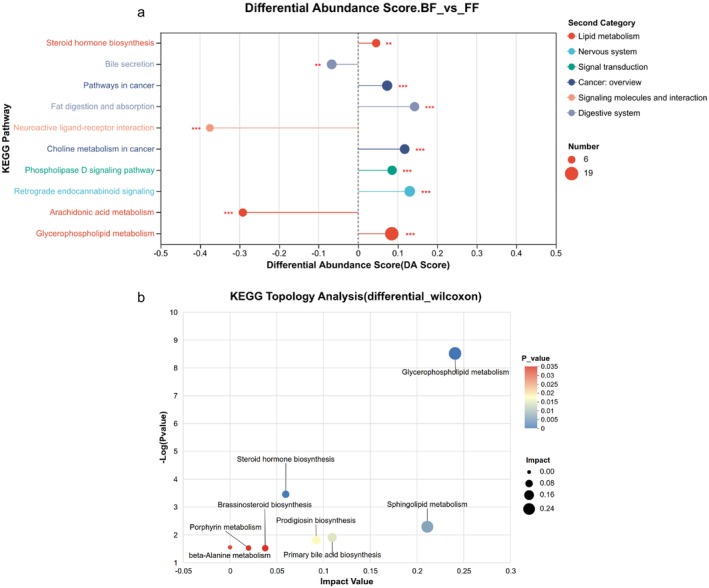
Kyoto Encyclopedia of Genes and Genomes database (KEGG) Pathway analysis of the identified differential metabolites between the two groups. (a) KEGG pathway differential abundance score plot. The DA Score reflects the overall changes of all metabolites in the metabolic pathway, with a score of 1 indicating that the expression trend of all annotated differential metabolites in the pathway is up‐regulated, −1 indicating that the expression trend of all annotated differential metabolites in the pathway is down‐regulated, and the length of the line segment represents the absolute value of the DA Score. The size of the dot indicates the number of differential metabolites annotated in the pathway, and the larger the dot indicates the greater the number of differential metabolites in the pathway. (b) KEGG topology analysis bubble plot. Each bubble in the figure represents a KEGG Pathway. The *x*‐axis indicates the magnitude of the Impact Value, which reflects the relative importance of metabolites within the pathway. The *y*‐axis shows the enrichment significance of metabolites involved in the pathway, expressed as log10(*p*‐value). The size of the bubble corresponds to the Impact Value, with larger bubbles indicating greater pathway importance. *p < 0.05, **p < 0.01, ***p < 0.001.

To further assess the global metabolic divergence between the two groups, we utilized the entire set of 600 differentially abundant metabolites (VIP > 1.0, *p* < 0.05) as the background for KEGG enrichment analysis and topology analysis. Among these, only79 significantly differential metabolites were mapped onto 190 different KEGG pathways (Table S3). 52 significantly enriched pathways (*p* < 0.05) were calculated by hypergeometric test, in which top 10 pathways included Glycerophospholipid metabolism, Arachidonic Acid (AA) metabolism, Retrograde endocannabinoid signaling, Choline metabolism in cancer, Phospholipase D signaling pathway, Neuroactive ligand‐receptor interaction, Fat digestion and absorption, Pathways in cancer, Bile secretion, Steroid hormone biosynthesis (Figure 4a). Compared with FF group, the overall expression of three pathways tend to be down‐regulated in BF group, including AA metabolism, Neuroactive ligand‐receptor interaction and Bile secretion, but the overall expression of other pathways tend to be up‐regulated in BF group (Figure 4a). A subsequent metabolic pathway topology analysis was performed to identify critical metabolic “hubs” by assessing their centrality and impact within the global network (Figure 4b). The analysis identified Glycerophospholipid metabolism as the most influential pathway with the highest impact value and statistical significance. Combined with the enrichment analysis, which showed a coordinated upregulation of lipid metabolism in BF infants. Detailed analysis revealed that 14 out of 19 identified differential glycerophospholipids, including phosphocholine (PC) (18:0/0:0), PC(16:0/0:0), Cytidine diphosphate‐diacylglycerol (CDP‐DG) (i‐24:0/i‐17:0), Phosphatidylserine (PS) (18:0/20:1(11Z)), Phosphatidylethanolamine (PE) (22:5(7Z,10Z,13Z,16Z,19Z)/20:1(11Z)), PS(20:2(11Z,14Z)/18:2(9Z,12Z)), Phosphatidyl‐N‐methylethanolamine (PE‐NMe) (20:5(5Z,8Z,11Z,14Z,17Z)/22:2(13Z,16Z)), PE‐NMe(22:6(4Z,7Z,10Z,13Z,16Z,19Z)/22:1(13Z)), PE(16:0/20:2(11Z,14Z)), PGP(18:1(9Z)/18:2(9Z,12Z)), PE(18:3(9Z,12Z,15Z)/20:0), Phosphatidylglycerophosphate (PGP) (18:1(9Z)/18:1(11Z)), Phosphatidylglycerol (PG) (18:1(11Z)/18:2(9Z,12Z)), and PG(18:2(9Z,12Z)/18:2(9Z,12Z)), were significantly upregulated in the BF group (Table S2).

**FIGURE 5:**
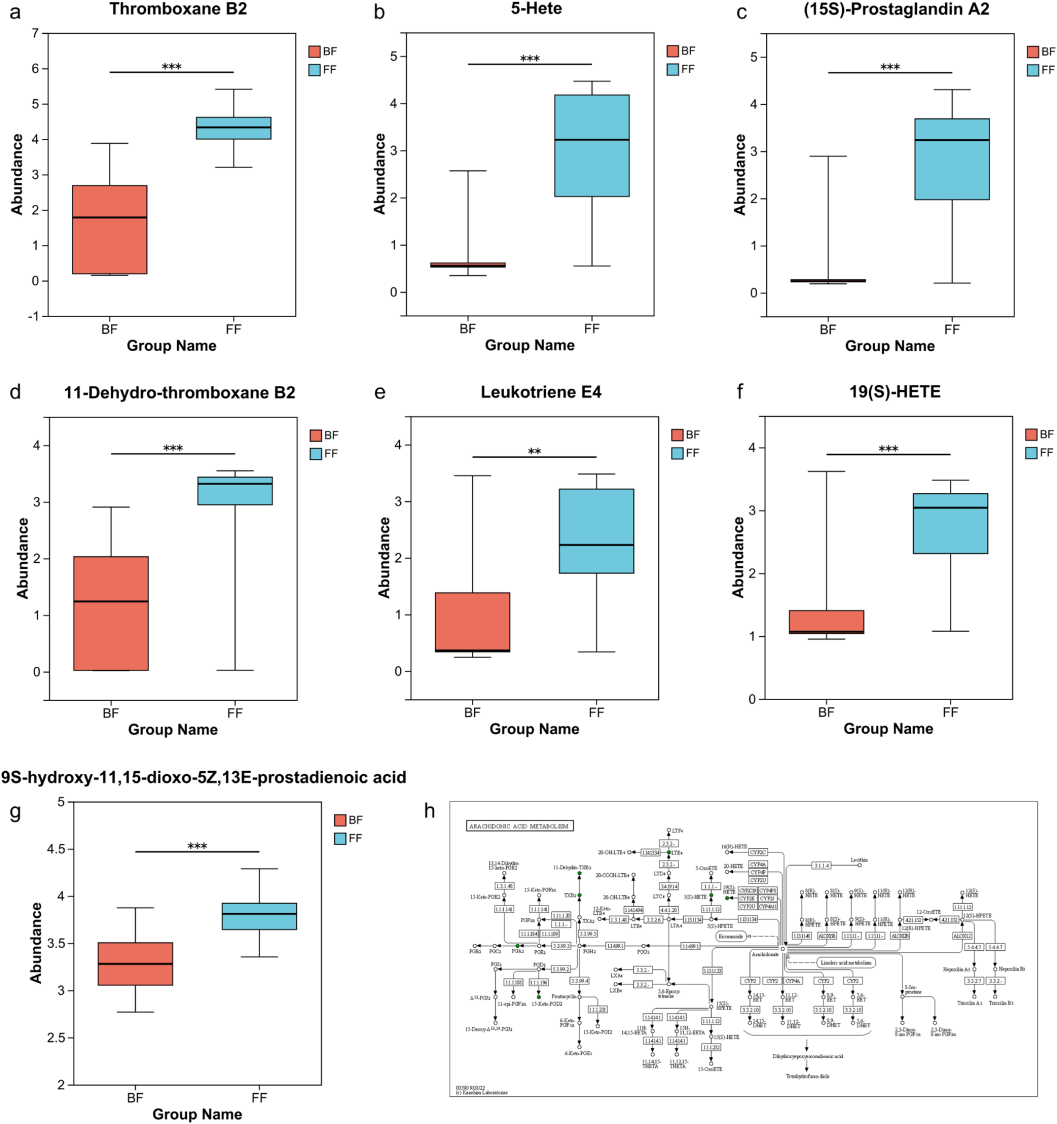
The analysis of significantly differential metabolites mapped onto Arachidonic acid (AA) metabolism pathway. Differential metabolites: (a) Thromboxane B2 (TXB2), (b) 5‐hydroxyeicosatetraenoid acid (HETE), (c) (15S)‐Prostaglandin A2(PGA2), (d) 11‐dehydro‐TXB2, (e) Leukotriene E4 (LTE4), (f) 19(S)‐HETE, (g) 9S‐hydroxy‐11,15‐dioxo‐5Z,13E‐prostadienoic acid. The *p* value was calculated by Wilcoxon rank‐sum test. **p < 0.01; ***p < 0.001. (h) The Schematic of the differential metabolites involved in AA metabolism. Green represents down‐regulated metabolites, and the open circles represent no change. 15‐Keto PGD2 is the traditional Name of 9S‐hydroxy‐11,15‐dioxo‐5Z,13E‐prostadienoic acid.

At the same time, further analysis of the significantly differential metabolites of top 100 VIP revealed that 12 metabolites could be mapped onto KEGG pathways (Table S2), including galactosylceramide (d18:1/18:1(9Z)), ginsenoside F1, 3a,21‐Dihydroxy‐5b‐pregnane‐11,20‐dione, TXB2, 5‐HETE, PGA2, 11‐Dehydro‐TXB2, Fructosyl‐lysine, CL(16:0/16:0/16:0/18:1(9Z)), 5‐Dehydroavenasterol, DG(8:0/17:0/0:0), and Enoxacin. The metabolites above were mostly mapped onto AA metabolism, including TXB2, 5‐HETE, PGA2, and 11‐dehydro‐TXB2, which indicated that AA metabolism is the metabolic pathway most significantly affected by feeding methods. In addition to these, the differential metabolites mapped onto the AA metabolism pathway also include Leukotriene E4 (LTE4), 19(S)‐HETE, and 9S‐hydroxy‐11,15‐dioxo‐5Z,13E‐prostadienoic acid (Figure 5).

### 3.4 The Correlations Between Gut Microbiota and Metabolites

**FIGURE 6:**
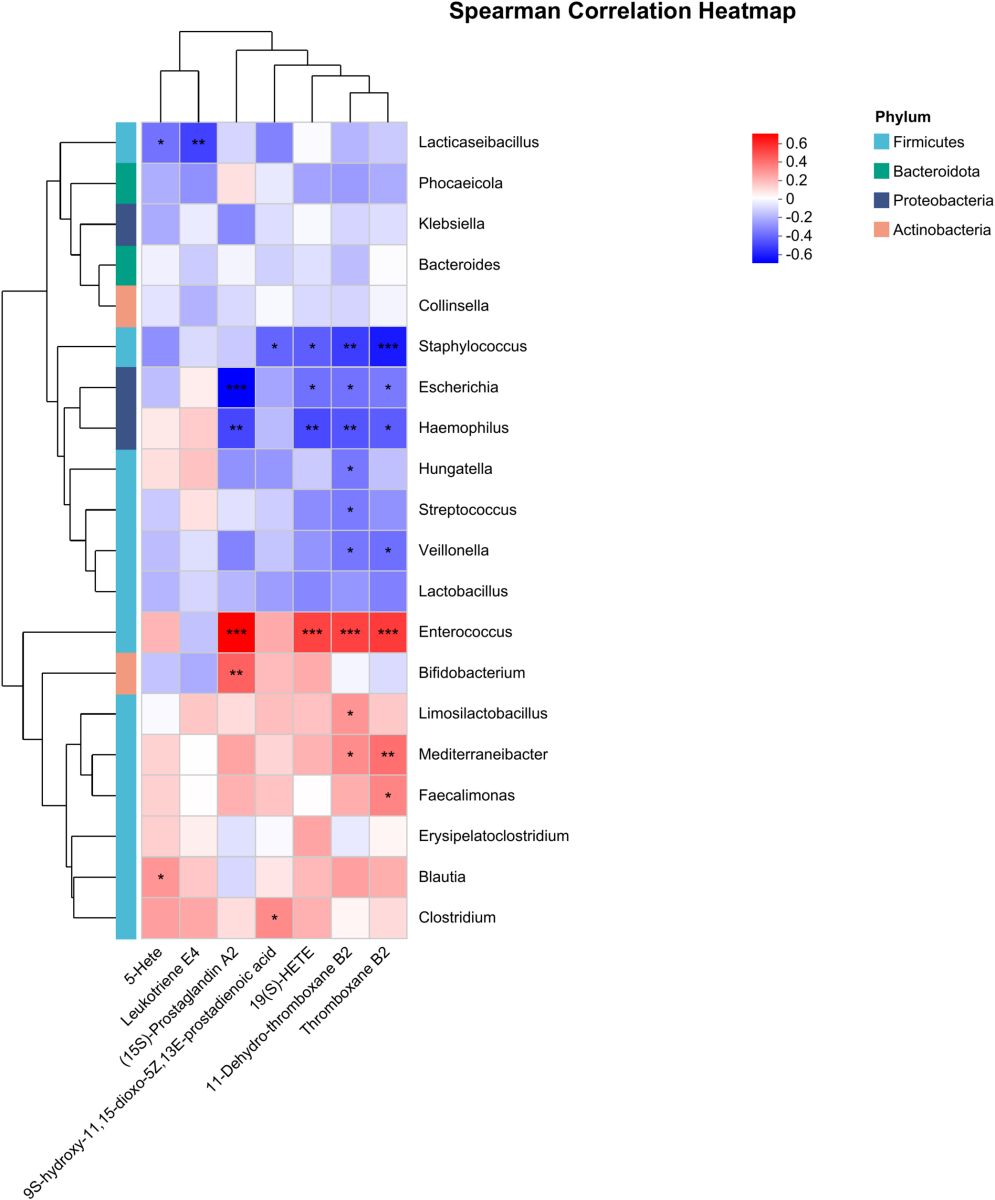
Heatmap diagram representing the correlation between the top 20 genera and specific metabolites in the two feeding methods. The *p* value was calculated by Spearman's rank correlation test, *p < 0.05, **p < 0.01, ***p < 0.001.

To delineate microbiota‐metabolite crosstalk within the AA metabolic axis, we computed Spearman correlations between the 20 most abundant genera (selected based on total relative abundance across all samples) and significantly differential metabolites annotated to the AA metabolism pathway (Figure 6). The results displayed that *Staphylococcus* was significantly negatively related to 9S‐hydroxy‐11,15‐dioxo‐5Z,13E‐prostadienoic acid, 19(S)‐HETE, 11‐Dehydro‐TXB2, and TXB2. *Escherichia* and *Haemophilus* were both significantly negatively related to PGA2, 19(S)‐HETE, 11‐Dehydro‐TXB2, and TXB2. *Enterococcus* was significantly positively related to PGA2, 19(S)‐HETE, 11‐Dehydro‐TXB2, and TXB2. *Mediterraneibacter* was significantly positively related to 11‐Dehydro‐TXB2 and TXB2.

## 4 Discussion

Primary microbial colonization during infancy orchestrates a developmental program that critically influences lifelong health trajectories (Healy et al. 2022; Shen et al. 2019; Thye et al. 2022; Uusitalo et al. 2016). Gut‐associated metabolites, which reflect the complex interplay between the microbiota, host, and dietary substrates, may represent pivotal mechanistic mediators linking early‐life nutrition to healthy development. In this study, to capture the evolving disparities in gut microenvironment between BF and FF infants, we updated the impact of feeding methods on infant gut microbiota and metabolites from 44 healthy infants and further reveal associations between key differential gut microbiota and metabolites that have been scarcely investigated in prior research.

Our results indicated significantly reduced microbial richness in BF group relative to FF group. This lower richness in BF infants likely reflects the selective pressure of human milk oligosaccharides (HMOs), which promotes a specialized microbiota dominated by beneficial taxa, a phenomenon consistent with prior studies (Bazanella et al. 2017; Ho et al. 2018; Stewart et al. 2018; Wood et al. 2018). At the phylum level, Proteobacteria abundance was significantly elevated in BF group, aligning with a prior observation (Odiase et al. 2023). This mirrors the predominance of Proteobacteria in breast milk microbiota (Pannaraj et al. 2017). At the genus level, *Escherichia, Staphylococcus, Haemophilus* and *Rothia* were more enriched in BF group, but *Enterococcus, Mediterraneibacter* were more enriched in FF group conversely, which are similar to previous studies (Di Guglielmo et al. 2022; Marrs et al. 2021). Utilizing the high resolution of PacBio sequencing, we further identified species‐level shifts that define the BF‐associated microbiome in this regional cohort. The BF group was characterized by a profound enrichment of *S. epidermidis* . Breast milk and the infant gut share key bacterial species, including *Escherichia* and *Staphylococcus*. These facultative anaerobes help reduce oxygen tension, facilitating the subsequent colonization of obligate anaerobes (Boudry et al. 2021). Notably, *S. epidermidis* is the most abundant species in breast milk (Ingram et al. 2024). Although *Escherichia* is one of the common pathogenic bacteria, studies have also shown that *Escherichia* demonstrate protective associations against atopic disorders, with reduction in infantile eczema incidence and lower risk of allergic colitis (Liu et al. 2017; Su and Kang 2022). *Staphylococcus* contributes to the saccharolytic fermentation of carbohydrates, and the end products generated exert beneficial effects on both host cells and the gut microbiota (Tanaka and Nakayama 2017). Children at high risk of developing asthma exhibited a marked reduction in the abundance of *Rothia* (Arrieta et al. 2015). *Mediterraneibacter* promotes inflammatory responses by increasing IL‐17A while decreasing IL‐10 levels of plasma (Huang et al. 2024). *Bifidobacterium* is typically considered the dominant genus in BF infants (Dinleyici et al. 2023), but our results showed no statistical difference in its abundance between the two groups. This phenomenon can be attributed to the high rate of prebiotic supplementation (GOS, FOS, and HMOs) in the formulas used by our FF cohort (88.2%), which has been shown to promote the proliferation of *Bifidobacterium* (Dinleyici et al. 2023; Sierra et al. 2015; Sims and Tannock 2020). This suggests that prebiotic supplementation may help narrow the gap in *Bifidobacterium* abundance between BF and FF infants. Collectively, our data delineated that while modern formula innovation, particularly prebiotic supplementation, can mimic the bifidogenic effect of breast milk, breastfeeding continues to exert a distinct modulation on the gut microbial ecosystem. Although often regarded as opportunistic, the BF‐associated microbiota can exhibit commensal or immunomodulatory roles in early life. Future studies with functional assays and longitudinal clinical follow‐up are needed to clarify the implications of this specific microbial signature.

Beyond microbial structural shifts, our untargeted profiling revealed that AA metabolism is the metabolic pathway most significantly affected by feeding methods, and the metabolites mapped onto this pathway, namely, TXB2, 5‐Hete, PGA2, 11‐dehydro‐TXB2, 19(S)‐HETE, and 9S‐hydroxy‐11,15‐dioxo‐5Z,13E‐prostadienoic acid were more enriched in FF group, in which TXB2, 11‐Dehydro‐TXB2, PGA2 and LTE4 are eicosanoids. These findings are consistent with a previous report that showed lower urinary tetranor‐prostaglandin D metabolite (t‐PGDM) excretion in BF versus FF infants at both one and 6 months of age (Shoji et al. 2018). Furthermore, our metabolic pathway topology analysis identified Glycerophospholipid metabolism as the most influential metabolic hub. Detailed analysis revealed that 14 out of 19 identified differential glycerophospholipids were significantly upregulated in the BF group. AA, a well‐established pro‐inflammatory mediator, is incorporated as a fatty acid chain in glycerophospholipids, embedded in the lipid bilayer structure of cell membranes, and its release is catalyzed by the regulated activity of phospholipase A2 (PLA2) (Dennis and Norris 2015). Free AA is metabolized through three principal enzymatic pathways: (a) Cyclooxygenase (COX) catalysis yielding prostanoids (TXs, PGs, prostacyclins); (b) Lipoxygenase (LOX; 5‐/12‐isoforms) generating LTs and HETEs; (c) Cytochrome P450 epoxygenase producing HETEs and epoxyeicosatrienoic acids (EETs) (Chiurchiù et al. 2018; Dennis and Norris 2015; Esser‐von Bieren 2017; Ferreira and Vane 1967; Samuelsson 1983; Samuelsson et al. 1975). These metabolites play a crucial role in modulating immune responses and inflammatory processes (Chiurchiù et al. 2018). TXB2 is involved in thrombus formation, inflammatory responses and increase vascular resistance by constricting blood vessels (Klotz et al. 1984). PGs prime eosinophil effector functions, thereby exacerbating type 2 inflammation in atopic disorders (Monneret et al. 2003). PGA2, the spontaneous dehydration product of PGE2, contributes to the development of Th1‐type immune responses (Thurnher et al. 2005). LTs, including cysteinyl LTs (CysLTs) and LTB4, possess well‐established pathophysiological roles across inflammatory disease spectra (Werz and Steinhilber 2006). As the terminal metabolite of CysLTs, LTE4 directly mediates asthmatic pathophysiology and serves as a robust biomarker for monitoring systemic CysLT biosynthesis (Montuschi and Peters‐Golden 2010; Montuschi et al. 2014; Chiu et al. 2014) established urinary LTE4 as a clinically validated biomarker strongly correlating with IgE‐mediated sensitization and subsequent development of allergic airway diseases after the age of 2.5‐HETE stimulates human eosinophil and neutrophil chemotaxis (Goetzl, Brash, et al. 1980; Goetzl, Weller, and Sun 1980; Valone et al. 1980), and induces human neutrophil degranulation (Stenson and Parker 1980). In studies on bronchial asthma, it has been found that 5‐HETE can induce tracheal contraction (Malo 1989). The simultaneous elevation of glycerophospholipids levels alongside the reduction of downstream AA metabolites in the BF group suggests that breastfeeding may stabilize these membrane lipid precursors or inhibit the enzymatic liberation of AA, thereby maintaining a low‐inflammatory metabolic tone in the infant gut. Taken together, our findings indicate that breastfeeding lowers the concentrations of metabolic products from the module of AA metabolism that have pro‐inflammatory effects. Therefore, it is indicated that breastfeeding can buffer the excessive inflammatory responses by inhibiting AA metabolism, conferring substantial protection against atopic diseases.

While the potential influence of dietary substrate on infant AA metabolism is acknowledged, evidence indicates that human milk AA concentration is homeostatically regulated and stable (0.47% ± 0.13% globally and 0.5%–0.65% across major Chinese cities), showing minimal response to maternal diet (Brenna et al. 2007; Richard et al. 2016; Salem Jr. and Van Dael 2020). In this study, the AA‐fortified formula (0.33%–0.61%) closely mirrored this physiological range. Despite comparable substrate availability, metabolic trajectories diverged markedly. These results suggest that, in the study, the differences in the fecal AA metabolites may not be primarily driven by dietary substrate availability. Given that gut metabolite profiles are shaped by the resident microbiota (Donia and Fischbach 2015; Nauta et al. 2013), we further performed correlation analyses to explore the potential interplay between the gut microbiota and these metabolic shifts. In line with this, our observations showed that Staphylococcus was significantly negatively related to 9S‐hydroxy‐11,15‐dioxo‐5Z,13E‐prostadienoic acid, 19(S)‐HETE, 11‐Dehydro‐TXB2 and TXB2. *Escherichia* and *Haemophilus* were both significantly negatively related to PGA2, 19(S)‐HETE, 11‐Dehydro‐TXB2 and TXB2. *Enterococcus* was significantly positively related to PGA2, 19(S)‐HETE, 11‐Dehydro‐TXB2 and TXB2. *Mediterraneibacter* was significantly positively related to 11‐Dehydro‐TXB2 and TXB2. These are not entirely consistent with previous studies. On the one hand, most of the previous studies have shown that eicosanoids are positively correlated with *Staphylococcus, Escherichia*, and *Haemophilus*. *S. epidermidis* can induce an increase in PGE2 and thromboxane synthesis (Gibson et al. 1988; Stout et al. 1994). *Escherichia coli* ( *E. coli* ) can promote the production of abundant PGs and LTs by macrophages (Werner et al. 2019). In addition, PGs could promote *E. coli‐*induced inflammatory responses via Toll‐like receptors (TLR) in macrophages (Gong et al. 2023; Khan et al. 2012). *Haemophilus influenzae* can induce PGE2 expression through TLR in lung tissues and the release of LTB4 from human neutrophils (Garofalo et al. 1991; Xu et al. 2008). On the other hand, although there are few studies demonstrating a direct association between eicosanoids and *Enterococcus*, some research has found that the trends in eicosanoids and *Enterococcus* are consistent (Fang et al. 2022; Mulyaningsih et al. 2010). The aforementioned studies are mostly in vitro studies or animal models that have identified direct mechanisms of association, which are pure results obtained by eliminating confounding factors. In contrast, this study is a clinical investigation that involves potential confounding factors that cannot be strictly controlled. The results presented here reflect the outcomes after a series of complex biochemical reactions in the gut microbiota. The gut microbiota can modulate fecal eicosanoid levels through several mechanistic pathways. Firstly, the gut microbiota can directly affect the activity of enzymes implicated in the biosynthesis and catabolism of eicosanoids, thereby altering their levels (Song et al. 2016). Secondly, the production of SCFAs by the gut microbiota may influence the pathways of eicosanoid synthesis and metabolism (Vinolo et al. 2011). Moreover, dysbiosis of the gut microbiota can exacerbate intestinal inflammatory responses and lead to increased levels of eicosanoids in feces (Videla et al. 1994). Eicosanoids can serve as signaling mediators that, in turn, modulate the composition and metabolic activity of the gut microbiota (Song et al. 2016). Therefore, the correlations observed between specific taxa and AA metabolites likely reflect the microbial modulation of this potent inflammatory axis, contributing to the lower inflammatory tone observed in BF infants.

Several methodological constraints warrant acknowledgment. Primarily, participant recruitment confined to a single geographic region may constrain extrapolation to populations with divergent dietary patterns and environmental exposures. The lack of detailed records on maternal dietary intake remains a significant confounder. Dietary substrate availability likely contributes to the observed metabolic profiles alongside microbial processing. Residual confounding from unmeasured variables, including perinatal antibiotic exposure and early‐life environmental triggers, could bias microbiome‐metabolite associations. Additionally, while our untargeted metabolomics approach provided a comprehensive overview of the metabolic landscape, the absence of validation through targeted metabolomics for key metabolites, especially specific lipid isomers, remains a technical limitation. This discovery‐based approach allowed us to capture a broad metabolic landscape, yet it necessitates cautious qualitative interpretation of specific lipid isomers. Future investigations should employ targeted metabolomics, utilizing Multiple Reaction Monitoring (MRM) and authentic chemical standards, to achieve absolute quantification and definitive identification of these eicosanoid species, thereby providing a more rigorous foundation for understanding their functional roles. Furthermore, the observational design and modest cohort size limit statistical power to detect moderate effects, potentially attenuating correlation robustness. To strengthen these findings, future investigations should implement multi‐center sampling across heterogeneous populations and incorporate prospective randomized feeding interventions. Crucially, longitudinal multi‐omics profiling across multiple time points is necessary to account for infant age as a primary developmental variable. Examining how the effects of feeding mode interact with chronological age will yield a more nuanced understanding of the dynamic maturation of immune function and gut ecology, ultimately establishing the causal pathways linking feeding modalities with microbial metabolic programming.

## 5 Conclusions

In conclusion, our study identifies a distinct gut microenvironment signature associated with breastfeeding at 6 months of age. We found that breastfeeding promotes a specific microbial assembly. While this profile deviates from the traditional paradigm of Bifidobacterium dominance, likely due to the high rate of prebiotic supplementation in our formula‐fed cohort which bridged the gap in bifidobacterial abundance, it reflects a unique ecological imprinting of breast milk on the early‐life gut environment. More importantly, significant disparities were observed in the fecal AA metabolic profile between the two groups, and breastfeeding was associated with a marked downregulation of pro‐inflammatory eicosanoids. These results suggest that breastfeeding may buffer excessive inflammation. Overall, these findings highlight that breastfeeding continues to exert a distinct immunological imprinting effect on infants by regulating the gut microenvironment. Rather than being universally beneficial, the observed BF‐associated microbiota represents a specific developmental stage that warrants further longitudinal investigation. Future mechanistic studies are necessary to decipher the complex crosstalk between breast milk constituents, these keystone microbiota, and the resulting immunomodulatory metabolites in shaping the developing immune system.

## Author Contributions

**Simin Zhu:** conceptualization, methodology, software, data curation, formal analysis, validation, visualization, writing – review and editing, writing – original draft, investigation. **Wenjuan Tu:** conceptualization, project administration, resources, supervision, funding acquisition, investigation. **Wenting Zhang:** conceptualization, methodology, formal analysis, validation, resources, writing – review and editing. **Li Qing:** conceptualization, investigation. **Chengan Wang:** conceptualization.

## Funding

This study was supported by the Jiangsu Provincial Medical Association Special Fund for Scientific Research (SYH‐32034‐0071); Scientific Research Project of Jiangsu Commission of Health (MQ2024030); Changzhou Sci&Tech Program (CJ20245049); Clinical Research Project of Nantong University (2025JZ014).

## Ethics Statement

The study was conducted in accordance with the Declaration of Helsinki, and approved by the Ethics Committee of Affiliated Changzhou Children's Hospital of Nantong University [Ethical Review of Medical Ethics at Changzhou Children's Hospital (Scientific Research) 2023‐023 date: 20 December 2023].

## Consent

Informed consent was obtained from all subjects involved in the study.

## Conflicts of Interest

The authors declare no conflicts of interest.

## Supporting information


**Figure S1:** OPLS‐DA permutation test bar chart. The horizontal axis represents the accuracy rate of the random model in the permutation test, the vertical axis represents the number of random models, the red bars indicate the count of Q^2^ values obtained from the permutation test, and the blue bars indicate the count of R^2^Y values obtained from the permutation test. The *p*‐value = (number of random models in the permutation test that outperform the original model) / (total number of random models in the permutation test). It is generally considered that the model is optimal when *p* < 0.05.

**Table S1:** OPLS‐DA model parameters.

**Table S2:** Identified differential metabolites between the two groups.

**Table S3:** KEGG pathway enrichment analysis of the identified differential metabolites between the two groups.

## Data Availability

All data relevant to the study are included in the article, or available from the corresponding author upon reasonable request. The raw full‐length 16S rRNA sequencing data have been deposited in the NCBI Sequence Read Archive (SRA) under BioProject accession number PRJNA1419559.
