# Supplementary material for: Integrated Analysis of Gut Microbiota and Metabolome Profiling Reveals the Effects of Feeding Approaches on Infants' Gut Microenvironment
Source: Food Sci Nutr. 2026 Apr 14;14(4):e71777. doi: 10.1002/fsn3.71777 (PMC13079968; doi:10.1002/fsn3.71777)
Supplement: Supplementary file 1 — Figure S1: OPLS‐DA permutation test bar chart. The horizontal axis represents the accuracy rate of the random model in the permutation test, the vertical axis represents the number of random models, the red bars indicate the count of Q2 values obtained from the permutation test, and the blue bars indicate the count of R2Y values obtained from the permutation test. The p‐value = (number of random models in the permutation test that outperform the original model) / (total number of random models in the permutation test). It is generally considered that the model is optimal when p < 0.05. [file FSN3-14-e71777-s003.docx]

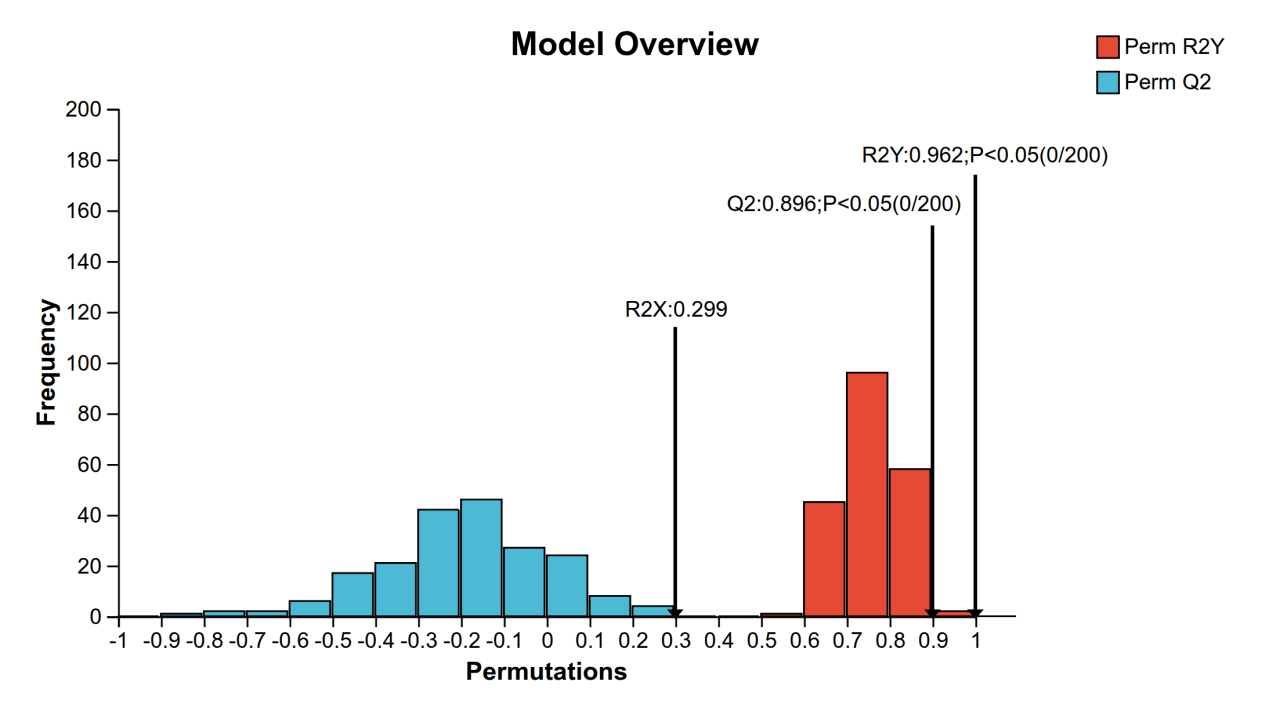


Figure S1 | OPLS-DA permutation test bar chart. The horizontal axis represents the accuracy rate of the random model in the permutation test, the vertical axis represents the number of random models, the red bars indicate the count of Q^2^ values obtained from the permutation test, and the blue bars indicate the count of R^2^Y values obtained from the permutation test. The *p*-value = (number of random models in the permutation test that outperform the original model) / (total number of random models in the permutation test). It is generally considered that the model is optimal when *p* < 0.05.
